# Usage Position and Virtual Keyboard Design Affect Upper-Body Kinematics, Discomfort, and Usability during Prolonged Tablet Typing

**DOI:** 10.1371/journal.pone.0143585

**Published:** 2015-12-02

**Authors:** Ming-I Brandon Lin, Ruei-Hong Hong, Jer-Hao Chang, Xin-Min Ke

**Affiliations:** 1 Department of Industrial and Information Management, National Cheng-Kung University, Tainan, Taiwan; 2 Department of Occupational Therapy, National Cheng-Kung University, Tainan, Taiwan; University of Perugia, ITALY

## Abstract

**Purpose:**

The increase in tablet usage allows people to perform computer work in non-traditional office environments. The aim of this study was to assess the effects of changes in tablet keyboard design on postures of the upper extremities and neck, discomfort, and usability under different usage positions during prolonged touch-typing.

**Methods:**

Eighteen healthy participants familiar with touch-screen devices were randomized into three usage positions (desk, lap, and bed) and completed six, 60-minute typing sessions using three virtual keyboard designs (standard, wide, split). Electrogoniometers continuously measured the postures of the wrists, elbow, and neck. Body discomfort and system usability were evaluated by questionnaires before and immediately after each typing session.

**Results:**

Separate linear mixed effects models on various postural measures and subjective ratings are conducted with usage position as the between-subject factors, keyboard design and typing duration as the with-in subject factors were conducted. Using the tablet in bed led to more extended wrists but a more natural elbow flexion than the desk position. The angled split virtual keyboard significantly reduced the extent of wrist ulnar deviation than the keyboard with either standard or wide design. However, little difference was observed across the usage position and keyboard design. When the postural data were compared between the middle and end of typing sessions, the wrists, elbow, and neck all exhibited a substantially increased range of joint movements (13% to 38%). The discomfort rating also increased significantly over time in every upper body region investigated. Additionally, the split keyboard design received a higher usability rating in the bed position, whereas participants had more satisfactory experience while using the wide keyboard in the traditional desk setting.

**Conclusions:**

Prolonged use of tablets in non-traditional office environments may result in awkward postures in the upper body that may expose users to greater risks of developing musculoskeletal symptoms. Adequate virtual keyboard designs show the potential to alleviate some postural effects and improve the user experience without changing the tablet form factors.

## Introduction

The popularity of tablets has blossomed dramatically since the introduction of the Apple iPad in 2010 as an alternative to traditional desktop and laptop computers. Approximately 230 million tablets were sold in 2014, and a recent survey indicated that approximately 53% of online adults in North America currently own a tablet [1, 2]. With the advent of the latest information technology, tablets show advantages for accomplishing a variety of computer-related work outside of the traditional office environment, including compactness, ease of use, and lightweight design. However, the compact design of tablets, with their display and input units integrated within a single flat touchscreen, appears to limit users’ flexibility in adjusting the relative positions of their upper bodies in conjunction with task requirements. Past studies have already shown that some features of laptop computers, such as the reduced keyboard size, fixed location of the internal input devices, and permanent connection of the display and keyboard, can increase wrist extension, elbow flexion, shoulder rotation, and head tilt in comparison to desktop computers [3–5]. Therefore, it is plausible that certain tablet interactions could also lead to awkward postures that result in substantial biomechanical stresses on human bodies. In addition, the existing ergonomics guidelines for workstation setup and input device designs (e.g. ANSI/HFES 100 and ISO-9241) may not be appropriate for tablet-related applications due to the considerable differences between tablets and traditional computers in form factor, interaction method, and usage environment. Therefore, there is an imminent need for studies examining the effects of those factors on the musculoskeletal outcomes and usability to aid in the development of ergonomics recommendations specifically for tablet usage.

Results from numerous epidemiological studies have suggested that long hours of computer work under non-neutral postures are associated with an increased risk of musculoskeletal symptoms or disorders (MSDs) in the neck and upper extremities [6, 7]. However, only limited research studies have thus far attempted to quantify those ergonomic risk factors in tablet usage [8–11]. It is believed that some mobile computing opportunities offered by tablets could cause users to assume working postures substantially different from those that have been extensively examined in past research [12]. Werth and Babski-Reeves [9] have suggested that typing on a tablet may lead to more extended and less ulnar-deviated wrist postures than typing on a notebook or a laptop computer. Awkward wrist postures, such as wrist extensions over 30 degrees or ulnar deviation over 15 degrees, are considered problematic because the resulting carpal tunnel pressure tends to exceed the 30 mmHg threshold proposed by Keir et al. [13]. Furthermore, the vertical location of tablet placement (on table surfaces or laps), the type of software tasks conducted, and its supported condition, all could influence how users vary their upper body postures during tablet interaction [9–11, 14]. For example, in a tablet study [11] involving a set of 3–5 minute simulated tasks (including internet browsing, movie watching, and email responding), the participants exhibited neck and head flexion levels generally greater than those reported in studies of desktop workstations [15] and laptop computing [3, 16, 17]. Recently, using a musculoskeletal modeling simulation, Vasavada et al. [8] further claimed that the gravitational demand on the neck musculature during tablet usage could be up to fivefold higher than that in the neutral posture. Meanwhile, few researchers have tried to examine the effect of tablet location on head and neck postures, as previous studies have shown that positioning a laptop on the lap causes more non-neutral neck postures [9, 16–18]. Nevertheless, the results were less consistent and confounded with the tablet tasks and supported cases examined in different studies [8, 9, 11].

To our knowledge, little to no data are readily available on the ergonomic exposure associated with prolonged use of tablets. The majority of published studies on tablet computing focus on the effects of form factors on body postures or muscle activities. Therefore, the simulated tasks performed in those studies tended to be completed within a short period, commonly ranging between 2 to 10 minutes for each experimental session [8, 10, 11, 14]. Some researchers have argued that one should be cautious in generalizing the conclusions from computer studies with short testing durations, as participants involved may adopt a posture that cannot be sustained for a long period of time, especially in non-traditional working environments [11, 17]. Besides, the potential differences across tablet configurations and work settings on musculoskeletal outcomes [12, 19] or task performance [20] may only become obvious when the usage duration is longer. For example, Lin et al. [21] observed muscular fatigue from participants’ finger extensors and flexors after two hours of a consecutive typing activity. Until now, how prolonged computer usage interacts with postural effects has not yet been evaluated in relation to tablet computing. This gap in the literature could be detrimental to the development of effective ergonomics guidance for mobile devices.

Plenty of research has been conducted to examine various geometric designs for physical keyboards in order to reduce the awkward wrist and forearm postures experienced while working with a computer [22–25]. For example, computer users with musculoskeletal disorders have shown improvement in their hand function and pain severity after several months of using some alternative split keyboard [26]. Recently, Trudeau et al. [14] examined different configurations of virtual keyboards for thumb typing in two-hand grip postures. Their participants expressed reduced discomfort when the split layout was used due to a lesser extent of thumb reaching movements and wrist extension. This may point to the potential role played by user interface design in alleviating the physiological loads experienced during tablet interaction. However, it is still unclear whether similar health benefits can be observed when a tablet is used for creation of typed documents with a more traditional two-handed typing approach outside of the conventional office environment.

Therefore, the objective of this study was to quantitatively determine the effects of changes in the usage position and virtual keyboard design on self-reported body discomfort, perceived usability, and postures of the wrist, elbow, and neck associated with a prolonged tablet-typing task. Based on past studies, we hypothesized that body discomfort and perceived usability of keyboard layouts would vary across the positions of tablet usage due to the different posture constraints and task demands imposed. Second, we hypothesized that the split keyboard layout would be effective in decreasing wrist deviation/extension and promoting more neutral elbow flexion, and thus lead to reduced discomfort in affected body regions. Third, we hypothesized that body discomfort would be increased over time and reflected as elevated variations in joint movements. Fourth, we hypothesized that the split keyboard design would be rated as having the highest perceived usability, regardless of the usage position.

## Materials and Methods

### Participants

Eighteen participants (nine men, nine women) in the age range of 20 to 31 years old were recruited to participate in this study. All participants were right-hand dominant, and had no history of MSDs over their neck, back, buttocks, or extremities. They also reported that they had not experienced any non-specific pain around the aforementioned body regions within last six months. In addition, they had not lifted any object over 5 kg over the past one week. They were able to touch-type at a speed over 35 words per minute (WPM). The typing speed and accuracy was confirmed by Typing Master Pro (v7.0, TypingMaster, Helsinki, Finland) during a 10-minute practice session on a 14-inch laptop (ThinkPad T410i, IBM, US). A summary of the participants’ demographic characteristics and typing skills is presented in Table 1. Male participants were generally taller and heavier than their female counterparts were. Otherwise, participants had a similar age and typing ability across genders. All participants owned and used smartphones with touch interfaces, and some of them had prior experience using tablets. Approval for this study was obtained from the Intuitional Review Board of the National Cheng Kung University (A-ER-101-042). Informed consent was obtained from all individual participants included in the study. Participants also gave consents for their photographs to be taken during the experimental sessions and later to be published in academic journals.

**Table 1:** Participant demographic characteristics and typing skills.

Variables	Male (n = 9)	Female (n = 9)
Age (years)	24.8 (±3.5)	23.1 (±0.9)
Height(cm)*	178.8(±5.5)	155.3(±4.2)
Weight (kg)*	71.6(±4.4)	53.8(±4.3)
Typing speed (WPM)	44.3 (±5.5)	45.5 (±7.6)
Typing accuracy (%)	95.9 (±2.3)	95.8 (±1.2)

Values are mean (± SD), WPM represents words per minute.

* Significant difference (p < 0.05) between male and female participants according to the results of Mann-Whitney U test

### Experimental design

This laboratory study adopted a 3 × 3 mixed factorial design with the usage position of the tablet, with typing as a between-subject factor and the design of the virtual keyboard as a within-subject factor. The participants were randomly assigned to one of three levels of usage position: DESK, LAP, or BED (to be described later). Based on the 10-min typing tests conducted on a desktop computer in the recruitment session, the results of Welch's test for analysis of variances indicated that there were no significant differences in either typing speed (F(2, 9.6) = 0.17, p = 0.847) or typing accuracy (F(2, 9.3) = 1.02, p = 0.397) among participants in three position groups. Each participant was required to conduct a series of 60-minute continuous typing tasks on an iPad tablet (iOS 5.1, Apple, Cupertino, CA, US), using all three different designs of virtual keyboard (STD, WIDE, and SPLIT) with two replicates (total of 360 minutes for each participant). The administration order of the six required typing sessions for each participant was randomly assigned to six non-consecutive days.

### Usage position

**Fig 1:**
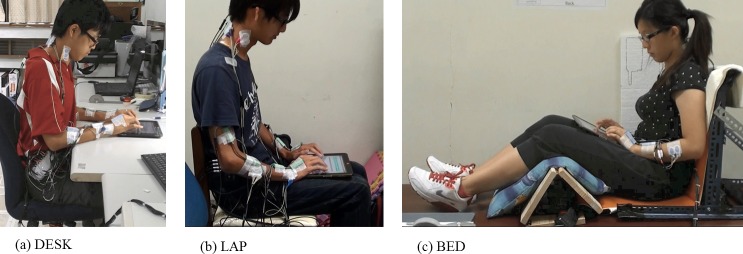
The three positions of tablet usage evaluated. (a) DESK position, participants seated on an adjusted chair with the tablet on the desk. (b) LAP position, participants seated on an adjusted chair with the tablet on the laps. (c) BED position, participants seated on a bed with knees bent and the tablet on the thighs.

For the first position (DESK), the participants assumed an upright sitting posture and performed the typing task with the tablet placed on top of a 74 cm high desk (Fig 1A). To reduce the variability associated with the seated posture and tablet location, the DESK position of tablet usage was setup based on the American National Standards Institute (ANSI) standards for a computer workstation (ANSI/HFES 100–2007). Before conducting the typing task, participants adjusted the height of the seat pan so that their thighs were parallel to the floor. The tablet was then placed in the landscape direction on the desk so that its central line matched with the midline of the participants’ trunks. During the typing task, the relative distance between the participants and the tablet was allowed to change in order to mitigate posture rigidity. However, over the course of the 60-minute experimental session (ISO 9241–5:1998), the elbow angle was required to remain between 70 and 135 degrees with the depth of the palm resting over or equal to 19 cm. Resting their forearms on the desk allowed the participants’ upper body weight to be partially supported, even when they preferred not to use the backrest of the chair. For the second position (LAP), participants sat on a height-adjustable chair with their backs against the backrest, the thighs approximately horizontal, the lower legs vertical, and the tablet evenly resting on theirs laps (Fig 1B). A chair without casters was used in the LAP position to reduce the chance of dropping the tablet. The third position (BED) required participants to sit on top of a firm bed (Fig 1C). A customized seat frame was employed to ensure that participants lie on the bed with their knees bent and their upper torso supported to simulate a position that is commonly used while interacting with a tablet on a bed or couch. In the beginning of the typing task, participants were instructed to place the tablet on top of their thighs with its bottom edge at the level of the umbilicus. However, they were permitted to move the tablet along their thighs during the typing session.

### Virtual keyboard design

**Fig 2:**
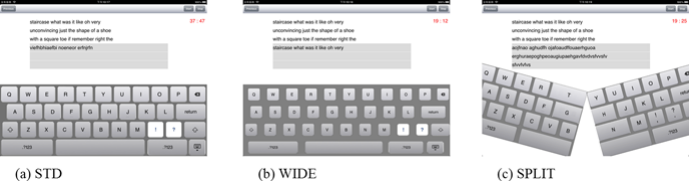
The three different designs of virtual keyboard evaluated. (a) STD design, the default layout provided in the tablet’s operation system. (b) WIDE design, the layout with broaden horizontal between-key spacing. (c) SPLIT design, the layout formed by two angled half keyboards

A customized typing APP with three virtual keyboard designs (STD, WIDE, and SPLIT) was created using Objective-C language (Xcode 4.3.1, Apple, Cupertino, CA, US). The touch screen of the tablet was separated into three areas; the text that was required to be typed was shown in the topmost area, three lines of text fields were located in the middle, and the virtual keyboard was accommodated in the rest of the screen area below the text fields (Fig 2). To eliminate the possible influences of learning effects and reading comprehension on the participants, the text to be typed in each session was randomly selected and reassembled from words in the book “The Lord of the Rings”, written by J. R. R. Tolkien. Therefore, the words in the text were not grammatically linked and had no punctuation marks. All three virtual keyboards used the same QWERTY layout, but the key size and the between-key spacing were varied. The STD design was identical to the stock QWERTY onscreen keyboard of the tablet with a key size of 14 mm in width by 15 mm in length. Considering the common ‘fat fingers’ problem in target positioning on touchscreen interfaces, the second virtual keyboard (WIDE) examined in the study was designed based on associated research and anthropometric measures of fingertips [27, 28]. The key size was set to 11 mm in width x 9 mm in length with a horizontal and vertical between-key spacing of 7 mm and 3 mm, respectively. Accordingly, the resulting 18 mm of horizontal center-to-center key spacing was in accordance with ISO 9241–410 standards for keyboards. The last angled SPLIT keyboard design was inspired by past research regarding physical keyboards in which splitting the keyboard into two halves promotes more neutral wrist posture by reducing ulnar deviation and wrist extension [22]. After separating the conventional QWERTY layout into halves through the right edge of the T, G, and B keys, the SPLIT virtual keyboard was formed by two rotating the half keyboards with an opening angle of 15 degrees between the halves [24].

### Apparatus

Wrist postures, along with the elbow and neck joint angles in the sagittal planes, were recorded by a flexible electro-goniometry system (MWX8, Biometrics, Gwent, UK). Due to their lightweight and unobtrusive design, strain-gauge electrogoniometers are a convenient and reliable way to examine joint kinematics in an ambulatory environment [29–31]. This system has been widely used in studies related to keyboard design and computer workstations [22, 25, 32]. The endblocks of the electrogoniometers (SG65 & SG110, Biometrics, Gwent, UK) were attached to participants’ skin by double-sided tape in a manner consistent with past studies and the manufacturer’s recommendations [29, 33]. Wrist flexion/extension and ulnar/radial deviation in the left and right hands were measured simultaneously by bi-axial electrogoniometers (SG65) affixed to the dorsum of the wrists in which one endblock was situated on the third metacarpal and the other on the midline of the forearm. The right elbow angles in the sagittal plane were recorded by an electrogoniometer (SG110) with two endblocks attached on the lateral midline of the forearm and upper arm, and the middle of the strain gauge located around the lateral epicondyle. The neck flexion/extension was also measured by an electrogoniometer (SG65) with the middle of the strain gauge positioned right above the spinous process of the 7^th^ cervical vertebra and two endblocks aligned along the upper and lower vertebrae. The participants were then asked to move their joints naturally to insure there was no hindrance from any attached electrogoniometers. The angular movements of the above joints were calibrated with respect to the reference values obtained in the neutral standing posture suggested by Kendall [34], and then sampled at a rate of 100 Hz throughout the typing task. The real-time data were displayed on a monitor for quality assurance and simultaneously recorded in a data logger for post-processing.

### Experimental protocol

The recruited participants were explained the experimental procedures in detail, and they gave their written informed consent before the study began. Time was allowed in the recruitment session for participants to practice and become familiar with the three virtual keyboard designs. Each participant was required to type in the assigned position with each virtual keyboard for 10 minutes. The participant was then scheduled to conduct his/her first formal 60-minute typing session two days later. The text passage displayed on the tablet for the recruitment session and formal sessions came from different book excerpts.

In the formal session, the participants were attached to all the electrogoniometers, and assumed a neutral standing posture for 10 seconds before beginning the formal 60-minute typing session. Postural data were continuously recorded by the flexible electrogoniometry system over the course of the experiment. A body map with ratings of perceived discomfort was presented to the participants before and after the typing session, as well as the moment right after 30 minutes of typing. The body map questionnaire allowed them to self-evaluate their discomfort level in each side in their wrists, forearms, shoulders, neck, upper and lower backs, and buttocks using Borg’s category ratio scale (CR-10)[35]. Participants were required to vocally respond to the experimenter’s inquiries regarding body discomfort level on a scale of 0 to 10 where a score of 0 corresponded to “nothing at all”, a score of 3 was “moderate”, and a score of 10 meant “extremely strong or almost maximum”.

After completing two replicates of each typing session with the same virtual keyboard, participants were instructed to answer another questionnaire, the System Usability Scale (SUS), to evaluate the usability of that specific keyboard design in respect to the given usage position. The SUS is considered one of the most widely used questionnaires for measuring perceptions of usability, and has been cited in more than 1,200 publications [36, 37]. It consists of 10 technology–neutral statements, to which users rate their level of agreement using a five-point scale from “strongly disagree” (1) to “strongly agree” (5). Since odd- and even-numbered statements are worded in opposite directions, a scoring technique is provided to convert and combine individual responses to obtain an overall SUS score within the range of 1 to 100. Work by Bangor et al. [36] and Sauro [38] both conclude that the SUS is a reliable and valid tool to assess user satisfaction and allows comparisons among a variety of products and services. Although the SUS was originally designed to be unidimensional, latest findings [39, 40] suggest that it may possess a bi-dimensional structure with correlated Learnable and Usable components. Borsci and et al. [39] have found that the obtained SUS ratings tend to be higher and exhibit the learnability dimension when respondents have enough experience of the product to be evaluated.

### Data management and statistical analysis

During the 60-minute typing session, two intervals of synchronized kinematics signals from the 10^th^ to the 25^th^ minute and the 55^th^ to the 60^th^ minute were used to calculate the 10^th^, 50^th^, and 90^th^ percentile (%) values of the amplitude probability distribution function (APDF). The APDF profiles have been commonly employed in physiological research to characterize musculoskeletal loading in various occupational tasks [18, 25, 41–43]. The 50^th^% (median) APDF angle was used as an indicator of the middle level of measured joint movements. Additionally, the range (extent of variation) of each joint movement (ROM), obtained from the difference between the 10^th^% and 90^th^% APDF was utilized to reflect posture adaptation due to the task requirements and the particular human-machine configuration [18, 19, 41].

The median angle and the range of joint movement were analyzed by four-way repeated measures analysis of variance (rANOVA) using linear mixed models. The within-subject factors were KEYBOARD with 3 levels (STD, WIDE, and SPILT) and TIME with 2 levels (10–25 min and 55–60 min). The between-subject factors were POSITION (DESK, LAP, and BED) and GENDER (MALE and FEMALE). These factors were treated as fixed effects and participants as a random effect in the rANOVA. For the omnibus F-tests, a value of p < 0.05 was considered statistically significant. Accordingly, the Tukey-Kramer method was applied for p-value adjustment in post-hoc multiple comparisons between levels of factors. If the POSITION by KEYBOARD interaction was significant, simple main effects were further examined. For example, the KEYBOARD effect was tested at different POSITION levels. Due to technical difficulties, few electrogoniometer data recorded during the last 5-minute of the 1-hour typing period was incomplete. Therefore, the rANOVA for individual joint measures in the present study were conducted based on the data from 209 of the total 216 typing sessions.

The SUS scores and the ratings of body discomfort were also analyzed by the same approach, except a TIME factor was not involved. It should be noted that the rANOVA for the SUS score was also performed through the aforementioned linear mixed modeling approaches because the data collected was not significantly deviated from normal judged from the result of Anderson-Darling normality rest (A-Squared = 0.416, p > 0.250). On the other hand, given the ordinal nature of the Borg CR-10 scale, the associated rANOVA for body regions were fitted using generalized linear mixed models by assuming the multinomial distribution of the response data with a cumulative logit link function [44]. Accordingly, differences between levels of the significant factors were evaluated by 95% confidence intervals of odds ratio estimates. All the aforementioned statistical analyses were conducted in SAS (v9.4, SAS Institute, Cary, NC, USA).

## Results

### Body joint angles

**Fig 3:**
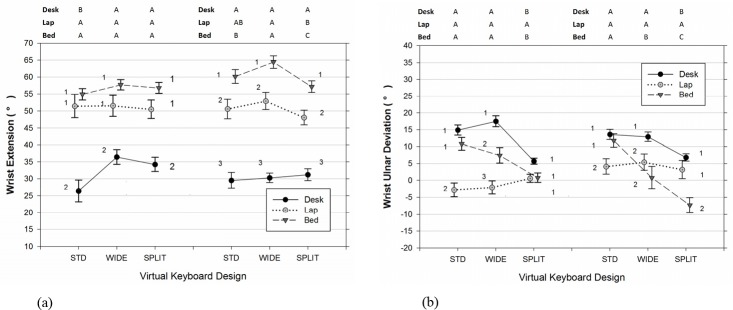
Wrist (a) extension and (b) deviation postures (50^th^ percentile) varied across different positions of tablet usage and virtual keyboard designs. Error bars represent +/- 1 SE. Negative values in (b) represent the wrist deviate toward the radial side. The conditions observed from the left (non-dominant) and right (dominant) wrist are separated accordingly in each sub figure. Conditions with the same number (1, 2, 3) denote groups without significant difference across the usage positions within the same virtual keyboard design. Conditions with the same character (A, B, C) denote groups without significant difference across the keyboard designs within the same usage position.

**Fig 4:**
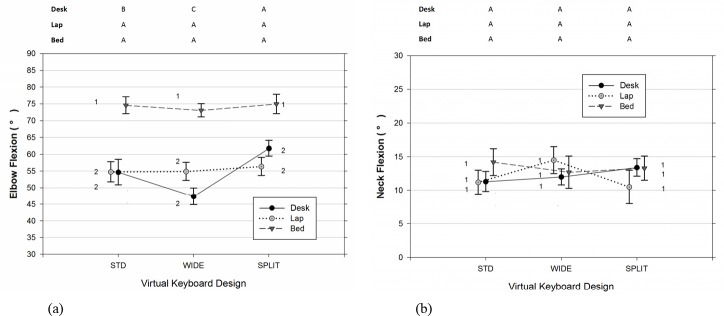
(a) Elbow and (b) neck postures (50^th^ percentile) varied across different positions of tablet usage and virtual keyboard designs. Error bars represent +/- 1 SE. Conditions with the same number (1, 2, 3) denote groups without significant difference across the usage positions within the same virtual keyboard design. Conditions with the same character (A, B, C) denote groups without significant difference across the keyboard designs within the same usage position.

**Fig 5:**
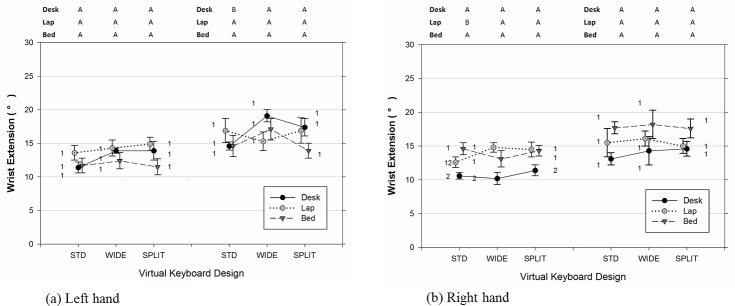
Range of wrist movement (APDF Range) in term of extension/flexion for the (a) left (non-dominant) and (b) right (dominant) hand varied across different positions of tablet usage and virtual keyboard designs. Error bars represent +/- 1 SE. The conditions observed from the early 10–25 min period (left side) and later 55–60 min period (right side) are separated accordingly in each sub figure. Conditions with the same number (1, 2, 3) denote groups without significant difference across the usage positions within the same virtual keyboard design. Conditions with the same character (A, B, C) denote groups without significant difference across the keyboard designs within the same usage position.

**Fig 6:**
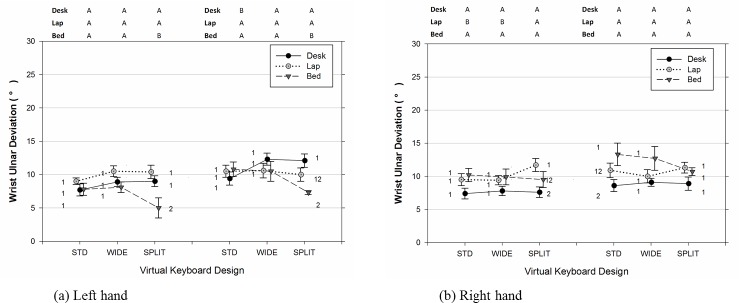
Range of wrist movement (APDF Range) in term of ulnar/radial deviation for the (a) left (non-dominant) and (b) right (dominant) hand varied across different positions of tablet usage and virtual keyboard designs. Error bars represent +/- 1 SE. The conditions observed from the early 10–25 min period (left side) and later 55–60 min period (right side) are separated accordingly in each sub figure. Conditions with the same number (1, 2, 3) denote groups without significant difference across the usage positions within the same virtual keyboard design. Conditions with the same character (A, B, C) denote groups without significant difference across the keyboard designs within the same usage position.

**Fig 7:**
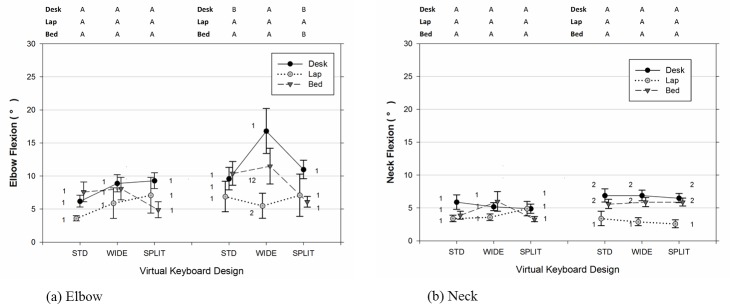
Range of wrist movement (APDF Range) in term of flexion/extension for the (a) elbow and (b) neck varied across different positions of tablet usage and virtual keyboard designs. Error bars represent +/- 1 SE. The conditions observed from the early 10–25 min period (left side) and later 55–60 min period (right side) are separated accordingly in each sub figure. Conditions with the same number (1, 2, 3) denote groups without significant difference across the usage positions within the same virtual keyboard design. Conditions with the same character (A, B, C) denote groups without significant difference across the keyboard designs within the same usage position.

As shown in Table 2, Fig 3, and Fig 4, there were significant differences in the joint angles of the upper limb and neck across usage positions and types of virtual keyboard employed. The data also indicated that participants significantly increased the range of joints movements examined when the duration of typing became lengthened (Figs 5–7). Moreover, the APDF range of wrist extension, wrist deviation, elbow flexion, and neck flexion in women tended to be wider than those observed in men (Table 2).

**Table 2:** Least-square mean (SE) of the postural parameters for rANOVA main effects Position, Keyboard, Time, Gender and p-values for the interactions ^1^ ^2^.

		Wrist Extension				Wrist Ulnar Deviation^3^				Elbow Flexion		Neck Flexion	
		Left		Right		Left		Right		Right		-	
		50th	APDF Range	50th	APDF Range	50th	APDF Range	50th	APDF Range	50th	APDF Range	50th	APDF Range
Position	p-value	**0.002**	0.433	**<0.001**	0.053	**<0.001**	0.115	**0.011**	0.078	**<0.001**	0.162	0.795	**0.024**
	DESK	32.4(4.0)^*B*^	15.0(1.0)^*A*^	30.3(3.2)^*B*^	12.4(1.0)^*B*^	12.7(1.9)^*A*^	9.9(0.6)^*A*^	11.1(1.8)^*A*^	8.2(0.9)^*A*^	54.6(3.2)^*B*^	10.3(1.5)^*A*^	12.2(1.4)^*A*^	6.0(0.6)^*A*^
	LAP	51.2(4.0)^*A*^	15.3(1.0)^*A*^	50.5(3.2)^*A*^	14.8(1.0)^*A*, *B*^	-1.4(1.9)^*B*^	10.2(0.6)^*A*^	4.2(2.0)^*A*, *B*^	10.5(0.9)^*A*^	55.4(3.1)^*B*^	6.0(1.5)^*A*^	12.0(1.6)^*A*^	3.5(0.6)^*B*^
	BED	56.3(3.9)^*A*^	13.6(1.0)^*A*^	60.6(3.2)^*A*^	16.0(1.0)^*A*^	6.8(2.0)^*A*^	8.3(0.6)^*A*^	2.1(2.0)^*B*^	11.2(0.9)^*A*^	74.7(3.1)^*A*^	8.0(1.5)^*A*^	13.4(1.7)^*A*^	5.2(0.6)^*A*, *B*^
Keyboard	p-value	**0.004**	**0.015**	**0.003**	0.640	**<0.001**	**0.031**	**<0.001**	0.758	**0.009**	**0.021**	0.752	0.501
	STD	44.4(2.4)^*B*^	13.8(0.6)^*B*^	46.9(1.9)^*A*, *B*^	14.1(0.6)^*A*^	7.6(1.3)^*A*^	9.2(0.4)^*A*, *B*^	9.9(1.4)^*A*^	10.0(0.5)^*A*^	61.3(2.1)^*A*, *B*^	7.4(1.0)^*B*^	12.1(1.2)^*A*^	4.9(0.4)^*A*^
	WIDE	48.4(2.4)^*A*^	15.3(0.6)^*A*^	49.2(1.9)^*A*^	14.4(0.6)^*A*^	7.7(1.3)^*A*^	10.1(0.4)^*A*^	6.3(1.4)^*B*^	9.8(0.5)^*A*^	58.8(2.1)^*B*^	9.4(1.0)^*A*^	13.1(1.2)^*A*^	5.1(0.4)^*A*^
	SPLIT	47.1(2.4)^*A*, *B*^	14.8(0.6)^*A*, *B*^	45.5(1.9)^*B*^	14.6(0.6)^*A*^	2.7(1.3)^*B*^	9.0(0.4)^*B*^	1.2(1.4)^*C*^	10.1(0.6)^*A*^	64.6(2.1) ^*A*^	7.6(1.0)^*A*, *B*^	12.4(1.2)^*A*^	4.7(0.4)^*A*^
Time	p-value	0.934	**<0.001**	0.218	**<0.001**	0.921	**<0.001**	0.747	**<0.001**	0.639	**<0.001**	0.580	**0.044**
	10–25 min	46.7(2.3)^*A*^	13.1(0.6)^*B*^	47.7(1.9)^*A*^	12.9(0.6)^*B*^	5.9(1.2)^*A*^	8.5(0.4)^*B*^	5.9(1.3)^*A*^	9.2(0.5)^*B*^	61.2(1.9)^*A*^	6.8(0.9)^*B*^	12.9(1.0)^*A*^	4.6(0.4)^*B*^
	55–60 min	46.6(2.4)^*A*^	16.2(0.6)^*A*^	46.6(1.9)^*A*^	15.8(0.6)^*A*^	6.0(1.2)^*A*^	10.4(0.4)^*A*^	5.6(1.3)^*A*^	10.7(0.5)^*A*^	61.9(2.0)^*A*^	9.4(0.9)^*A*^	12.2(1.1)^*A*^	5.2(0.4)^*A*^
Gender	p-value	0.933	**0.007**	0.493	0.438	0.327	**<0.001**	**<0.001**	**0.011**	0.252	**<0.001**	**0.011**	**0.031**
	Female	46.4(3.2)^*A*^	16.2(0.8)^*A*^	45.9(2.6)^*A*^	14.8(0.8)^*A*^	7.1(1.6)^*A*^	10.8(0.5)^*A*^	10.3(1.6)^*A*^	11.2(0.7)^*A*^	63.7(2.6)^*A*^	11.1(1.2)^*A*^	10.3(1.3)^*B*^	5.6(0.5)^*A*^
	Male	46.8(3.2)^*A*^	13.1(0.8)^*B*^	48.4(2.6)^*A*^	13.9(0.8)^*A*^	4.9(1.6)^*A*^	8.1(0.5)^*B*^	1.2(1.6)^*B*^	8.7(0.7)^*B*^	59.5(2.6)^*A*^	5.1(1.2)^*B*^	14.8(1.3)^*A*^	4.1(0.5)^*B*^
Interactions													
Position x Keyboard	p-value	**0.019**	**0.008**	0.075	0.494	**<0.001**	**<0.001**	**<0.001**	**0.016**	0.054	**<0.001**	0.383	0.184
Position x Time	p-value	0.830	0.217	0.962	0.085	0.696	**0.008**	0.998	0.052	0.978	0.123	0.827	**<0.001**
Keyboard x Time	p-value	0.903	0.473	0.800	0.620	0.988	0.924	0.935	0.251	0.911	0.275	0.928	0.473
Gender x Time	p-value	0.896	0.315	0.711	0.736	0.652	0.897	0.722	0.970	0.474	0.057	0.967	0.906

1 values are in degrees. Repeated measures ANOVA with Participants as a random effect and Position, Keyboard, Time, and Gender as fixed effects.

2 For each main dependent variable, the groupings based on the host hoc analyses are indicated by the superscript and ranked in a descending order such as A > B > C. The same superscript means no significant difference.

3 Negative values represent the wrist deviate toward the radial side.

Both the position of tablet usage and virtual keyboard design had a significant influence on the medial wrist extension (for left wrist: Position [F(2, 14) = 9.99, p = 0.002], Keyboard [F(2, 179) = 5.78, p = 0.004]; for right wrist: Position [F(2, 14) = 23.09, p < 0.001], Keyboard [F(2, 179) = 5.97, p = 0.003]). While typing in bed, participants experienced the most extended wrist postures, more than 50 degrees in the 50^th^ percentile level, followed by on the lap and on the desk (Table 2). Besides, as seen in Fig 3A, participants’ right hands (M = 60.6, SE = 3.5) and left hands (M = 56.5, SE = 3.5) exhibited dissimilar Figwrist extension level in the BED position, t(385) = 4.04, p < 0.001. Post-hoc analyses in Table 2 for the 50^th^ percentile measure further revealed that the use of the WIDE keyboard generally led to a higher wrist extension than either the STD keyboard (for left wrist: t(179) = 3.34, p = 0.003; for right wrist: t(179) = 2.13, p = 0.087) or SPLIT keyboard (for left wrist: t(179) = 1.14, p = 0.494; for right wrist: t(179) = 3.43, p = 0.002). Moreover, both wrists demonstrated, on average, a 3 degree wider ROM toward the end of the 60-minute typing session, compared to the earlier 10 to 25 minute period(for left wrist, Fig 5A: [F(1, 179) = 56.38, p < 0.001]; for right wrist, Fig 5B: [F(1, 179) = 42.24, p < 0.001]).

As expected, the virtual keyboard design, position of tablet usage, and their interactions had significant effects on the posture of wrist deviation on both hands (Table 2). Specifically, the use of the angled SPLIT keyboard significantly reduced the overall wrist ulnar deviation compared to the other two keyboard designs (for left wrist: SPLIT vs STD, t(179) = 4.65, p < 0.001; SPLIT vs WIDE, t(179) = 4.86, p < 0.001) (for right wrist: SPLIT vs STD, t(179) = 6.45, p < 0.001; SPLIT vs WIDE, t(179) = 3.89, p < 0.001). According to the results of analysis of simple main effects on the 50^th^ percentile measure of wrist deviation, the wrist postures observed while typing on the SPLIT design were also more similar across the three examined working positions, particularly for the left wrist, F(2, 179) = 1.82, p = 0.165. It should be noted that, as shown in Fig 3B, using the SPLIT keyboard in the BED position did cause participants significantly deviate their right wrists toward its radial side, compared to the other two usage positions (for BED vs DESK: t(179) = 4.50, p < 0.001; for BED vs LAP, t(179) = 2.96, p = 0.004). In addition, the rANOVA results from Table 2 suggested that participants working in the DESK position had greater ulnar deviation than those either working in the LAP position (for left wrist: t(14) = 5.44, p < 0.001) or in the BED position (for right wrist: t(14) = 3.62, p = 0.007). To further quantify the influence of hand dominance on usage position and keyboard design in Tablet typing, additional rANOVA with a new Side factor and its interaction terms was performed. The results indicated that LAP position participants, on average, postured their right wrists 6 degrees more deviated toward the ulnar side, compare to the left wrist, t(385) = 3.59, p < 0.001. On the contrary, in the BED position a higher level of ulnar deviation was observed in participants’ left wrists in comparison to their right ones, t(385) = 3.22, p = 0.001. When examining the time effect, we found a significant increase in the range of wrist movement during the last 5-minute typing period, irrespective of the virtual keyboard design employed (Table 2). Nevertheless, both Fig 6 and the further analyses of simple Time main effect with respect to usage position all suggested that the increase of the APDF range in wrist deviation over time was less obvious in the LAP position for both hands (for left wrists: [F(1,179) = 0.45, p = 0.503]; for right wrists: [F(1,179) = 1.47, p = 0.227]).

Similarly, elbow flexion was significantly influenced by the position of tablet usage and the keyboard design at the 50^th^ percentile level (Position [F(2, 14) = 13.33, p < 0.001]; Keyboard [F(2, 179) = 4.88, p = 0.009]). Participants in the BED position inclined to maintain approximately 19 degrees greater elbow flexion, compared to the angles observed from the other two positions (for BED vs DESK, t(14) = 4.50, p = 0.001; for BED vs LAP, t(14) = 4.43, p = 0.002). Meanwhile, the results of post-hoc analyses in Table 2 also indicated that elbow data from the SPLIT virtual keyboard tended to be higher than those from the WIDE design, t(179) = 3.11, p = 0.006, particularly in the DESK position (see Fig 4A). Similar to the wrist postures, participants varied more in the range of elbow separation when the typing task was longer. As shown in Table 2, the APDF range of elbow flexion recorded between the duration of the 55^th^ to 60^th^ minutes was, on average, 38% higher than those between the 10^th^ to 25^th^ minute periods. This phenomenon was particularly obvious from those required to touch-type continuously in the DESK and BED positions with F(1,179) = 5.84, p = 0.017 and F(1, 179) = 12.01, p < 0.001, respectively (also see Fig 7A).

The position of tablet usage had a small influence on the median level of neck flexion, as well as the design of virtual keyboard (Fig 4B). However, there was a significant difference in the ROM of neck flexion across the different working positions, F(2,14) = 4.92, p = 0.024. Compared to typing in the LAP position, the results of post-hoc analyses in Table 2 suggested that typing in the DESK position resulted in a higher APDF range of neck flexion, t(14) = 3.07, p = 0021. The overall time effect, as well as its interaction with the position, was significant when the neck flexion was analyzed in terms of the movement range with F(1, 179) = 4.10, F = 0.044 and F(2, 179) = 9.09, p < 0.001, respectively. As shown in Fig 7B, participants in either the DESK (F(1, 179) = 6.68, p = 0.011) or BED position (F(1, 179) = 5.32, p = 0.022) were observed to flex their necks in a wider range when the time approached the end of the 60-minute typing session.

### Perceived body discomforts

Table 3 presents the results of statistical analyses of the subjective discomfort ratings to pronged tablet typing across a variety of combinations of working positions and keyboard design. First, the self-perceived body discomforts, as expected, significantly increased with time in all body regions evaluated, including the upper extremities, back, neck, shoulders, and the buttocks (left wrist [F(2, 293) = 75.35, p < 0.001]; right wrist [F(2, 294) = 55.76, p < 0.001]; left arm [F(2, 292) = 57.21, p < 0.001]; right arm [F(2, 294) = 54.57, p < 0.001]; shoulder [F(2, 292) = 83.64, p < 0.001]; neck [F(2, 292) = 77.93, p < 0.001]; upper back [F(2, 292) = 60.40, p < 0.001]; lower back [F(2, 294) = 49.50, p < 0.001]; buttocks [F(2, 292) = 33.71, p < 0.001]). Moreover, the data also suggested there were significant interactions between time and position in the left wrist/arm, shoulder/neck, and buttock region. Secondly, the usage position substantially influenced the feeling of discomfort in all body regions (Table 3). In particular, typing in the LAP position troubled subjects’ necks, shoulders, upper back, and buttocks, with an average discomfort rating approaching or above the “moderate” (i.e. 3) level. On the contrary, the traditional DESK setting received the lowest discomfort rating for all body regions. Lastly, the virtual keyboard design also significantly affected the perceived body discomfort in the wrists, arms, and shoulders region (left wrist [F(2, 293) = 6.56, p = 0.002]; right wrist [F(2, 294) = 3.55, p = 0.030]; left arm [F(2, 292) = 7.31, p < 0.001]; right arm [F(2, 294) = 5.24, p = 0.006]; shoulder [F(2, 292) = 5.73, p = 0.004]). As can been seen in Table 3, the results of further post-hoc analyses based on the 95% confidence limits of odd ratio estimates from those body areas generally suggest that the SPLIT virtual keyboard seemed to be the most comfortable design, followed by the WIDE keyboard and the STD one. However, none of the examined interactions between the keyboard and time effects was significant.

**Table 3:** Mean (SE) of body discomfort ratings in Borg CR-10 scale for rANOVA main effects Position, Keyboard, Time, Gender and p-values for the interactions ^1^ ^2^.

		Wrist		Arm		Shoulder	Neck	Upper back	Lower back	Buttocks
		Left	Right	Left	Right	-	-	-	-	-
Position	p-value	**< 0.001**	**< 0.001**	**0.044**	**< 0.001**	**< 0.001**	**< 0.001**	**< 0.001**	**< 0.001**	**< 0.001**
	DESK	1.3(0.1)^*B*^	0.7(0.1)^*B*^	1.5(0.2)^*B*^	0.8(0.1)^*B*^	1.2(0.1)^*C*^	1.4(0.1)^*C*^	0.7(0.1)^*C*^	0.5(0.1)^*C*^	0.3(0.1)^*C*^
	LAP	2.1(0.1)^*A*^	1.8(0.1)^*A*^	1.7(0.2)^*A*^	1.6(0.2)^*A*^	3.1(0.1)^*A*^	3.1(0.1)^*A*^	2.7(0.2)^*A*^	2.3(0.1)^*A*^	2.9(0.2)^*A*^
	BED	1.8(0.1)^*A*^	2.0(0.1)^*A*^	1.4(0.1)^*A*, *B*^	1.6(0.1)^*A*^	2.3(0.2)^*B*^	2.4(0.2)^*B*^	2.1(0.2)^*B*^	1.6(0.1)^*B*^	1.8(0.2)^*B*^
Keyboard	p-value	**0.002**	**0.030**	**< 0.001**	**0.006**	**0.004**	0.067	0.215	0.090	0.574
	STD	2.2(0.1)^*A*^	1.7(0.1)^*A*^	2.0(0.1)^*A*^	1.6(0.1)^*A*^	2.6(0.1)^*A*^	2.6(0.1)^*A*^	2.0(0.1)^*A*^	1.7(0.1)^*A*^	1.7(0.1)^*A*^
	WIDE	1.7(0.1)^*A*, *B*^	1.5(0.1)^*A*, *B*^	1.5(0.1)^*A*^	1.3(0.1) ^*B*^	2.1(0.1)^*B*^	2.3(0.1)^*A*^	1.9(0.1)^*A*^	1.4(0.1)^*A*^	1.6(0.1)^*A*^
	SPLIT	1.3(0.1)^*B*^	1.3(0.1)^*B*^	1.2(0.1)^*B*^	1.1(0.1)^*B*^	1.9(0.1)^*B*^	2.0(0.1)^*A*^	1.6(0.1)^*A*^	1.3(0.1)^*A*^	1.6(0.1)^*A*^
Time	p-value	**< 0.001**	**< 0.001**	**< 0.001**	**< 0.001**	**< 0.001**	**< 0.001**	**< 0.001**	**< 0.001**	**< 0.001**
	Begin	0.2(0.1)^*C*^	0.2(0.1)^*C*^	0.3(0.1)^*C*^	0.3(0.1)^*C*^	0.5(0.1)^*C*^	0.6(0.1)^*C*^	0.3(0.1)^*C*^	0.2(0.1)^*C*^	0.2(0.1)^*C*^
	Middle	2.0(0.1)^*B*^	1.7(0.1)^*B*^	1.7(0.1)^*B*^	1.5(0.1)^*B*^	2.5(0.1)^*B*^	2.6(0.1)^*B*^	2.1(0.1)^*B*^	1.6(0.1)^*B*^	1.8(0.1)^*B*^
	After	3.0(0.1)^*A*^	2.5(0.1)^*A*^	2.6(0.1)^*A*^	2.2(0.1)^*A*^	3.7(0.1)^*A*^	3.7(0.1)^*A*^	3.2(0.1)^*A*^	2.5(0.1^*)A*^	2.9(0.1)^*A*^
Gender	p-value	0.173	**0.029**	0.252	**0.023**	0.586	0.902	0.144	0.500	0.437
	Female	1.6(0.1) ^*A*^	1.6(0.1) ^*A*^	1.3(0.1) ^*A*^	1.4(0.1) ^*A*^	2.0(0.1) ^*A*^	2.1(0.1) ^*A*^	1.8(0.1) ^*A*^	1.4(0.1) ^*A*^	1.6(0.1) ^*A*^
	Male	1.9(0.1) ^*A*^	1.3(0.1) ^*B*^	1.7(0.1) ^*A*^	1.2(0.1) ^*B*^	2.4(0.1) ^*A*^	2.4(0.2) ^*A*^	1.8(0.1) ^*A*^	1.5(0.1) ^*A*^	1.7(0.1) ^*A*^
Interactions										
Position x Keyboard	p-value	0.094	0.140	0.260	0.313	0.122	0.249	0.096	0.099	0.517
Position x Time	p-value	**0.011**	0.264	**0.018**	0.208	**0.009**	0.288	0.205	0.158	**< 0.001**
Keyboard x Time	p-value	0.503	0.523	0.846	0.985	0.464	0.625	0.391	0.606	0.802
Gender x Time	p-value	0.337	0.788	**0.017**	0.278	**< 0.001**	**< 0.001**	**0.030**	**0.012**	**< 0.001**

1 values are in degrees. Repeated measures ANOVA with Participants as a random effect and Position, Keyboard, Time, and Gender as fixed effects.

2 For each main dependent variable, the groupings based on the host hoc analyses are indicated by the superscript and ranked in a descending order such as A > B > C. The same superscript means no significant difference.

### Subjective usability ratings

**Fig 8:**
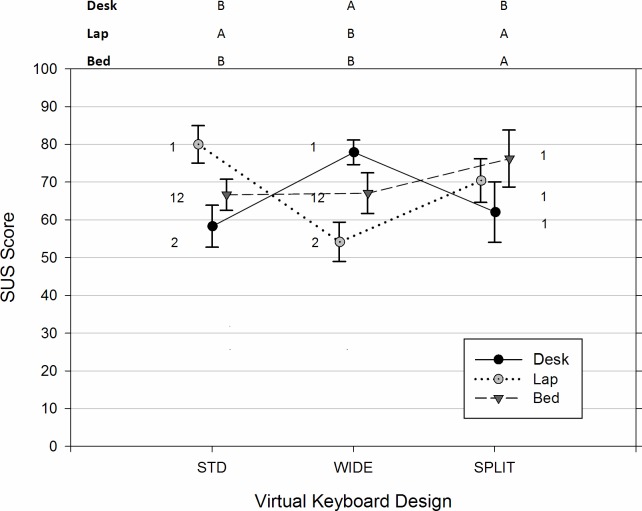
Mean perceived usability of virtual keyboard designs varied across different positions of tablet usage. Conditions with the same number (1, 2, 3) denote groups without significant difference across the usage positions within the same virtual keyboard design. Conditions with the same character (A, B, C) denote groups without significant difference across the keyboard designs within the same usage position.

The results of rANOVA implied that the position of tablet usage affected the overall perceived ratings of usability through significant Posture by Keyboard interaction, F(4, 24) = 8.31, p < 0.001. As can been seen in Fig 8, in the traditional DESK setting, participants gave the WIDE keyboard the highest SUS score (M = 77.9, SE = 3.3) among the three keyboard designs, F(2, 24) = 5.20, p = 0.013. In contrast, both the angled SPLIT and the STD virtual keyboard were rated preferable to the WIDE one when the tablet was used in the LAP position, F(2, 24) = 9.39, p < 0.001. Likewise, in the BED position, the SPLIT keyboard had a significantly greater perceived usability (M = 76.3, SE = 7.6) than the STD and WIDE designs, F(2, 24) = 4.50, p = 0.022. In addition, the simple main effects analyses also revealed that, unlike the other two virtual keyboards (STD [F(2, 24) = 4.13, p = 0.029; WIDE [F(2, 24) = 4.84, p = 0.0170]), the usability perceived by participants regarding the SPLIT keyboard design was not significantly affected by the working position of tablet usage, F(2, 24) = 2.14, p = 0.140.

## Discussion

As the use of tablets becomes more prevalent due to the advance of mobile computing technology, it is important to understand the potential adverse health effects of prolonged use of its virtual keyboard on the musculoskeletal system on humans in various environments. The major findings of this study were that typing on a tablet outside the office or work setting generally leads to more non-neutral postures and greater neuromuscular discomfort. Moreover, the body discomfort and variation in the upper-body joint movements tend to increase as the typing duration extends. However, some of these negative health consequences could be partially mitigated through adequate virtual keyboard design. A virtual keyboard design with a higher perceivable usability tended to result in a less awkward and more comfortable typing posture. There is, however, no single universal solution for tablet typing because of the substantial varied physiological strains imposed by the usage positions assumed in various working environments.

### Wrist Extension

This study suggests that the position of the tablet affected how users interacted with a virtual keyboard while typing. Touch-typing on a tablet in either the BED or LAP position resulted in significantly higher wrist extensions in both hands compared to the DESK position, a more common working posture in office environments. Other researchers also found that conducting finger-typing tasks with a tablet supported by a surface lower than elbow-level leads to a higher mean wrist extension [9, 10]. Nevertheless, in the work of Young et al. [10], the reported 35 degrees and 25 degrees of mean wrist extension with the tablet on a table and laps, respectively, are lower than the values observed in the present study (Fig 3A). This discrepancy could be attributed to the difference in the experimental designs, as the tablets used for the email task in the prior study were placed in cases with tilt angles ranging from 15 to 45 degrees, and the typing lasted for only 3 minutes. In addition, the varied dimensions of the backrest and supported surface of sitting objects might promote users to assume dissimilar sitting postures (e.g. forward vs. reclined), and consequently alter the forearm support and wrist posture accordingly [17]. We observed greater than 26 degrees of median wrist extension in all examined configurations. This falls into the upper end of the spectrum of values reported in past studies of VDT workstations, physical keyboard designs, computing tasks, and laptop computers [3, 4, 17, 22–24, 41, 45, 46]. Only a few studies that required participants to type on a physically split keyboard with extreme slopes [47], or use a laptop computer placed on the lap [16] have reported mean wrist extension values higher than 35 degrees. The relatively greater median wrist extension observed in tablet studies may be partly accounted for by the need to float fingers in the air to avoid unintentional key activation, or the need of visually assess the key locations due to the lack of tactile feedback [10, 32]. In addition, the problem of visual obstruction could be further exaggerated given the limited dimensions of the virtual keyboard compared with its full-sized physical counterpart. Considering that most participants in the BED position also had a tendency to use their wrists to stop the tablet from sliding, collectively, the aforementioned reasons may account for the extremist wrist extensions observed in that particular posture.

Our experimental data also indicated that the effect of reducing wrist extension by using the angled split virtual keyboard was not unanimous, but instead limited to the BED and LAP positions. In these two positions where users were prone to extend their wrists further, in the right wrist, the standard virtual keyboard required approximately 3 more degrees of median wrist extension than the angled split layout (Fig 3A). This difference was slightly less than the 4–5 degree value reported by Marklin et al. [22], in which they compared the physical split fixed-angle keyboard and its conventional counterpart in an office setting. The nature of touchscreen technology requires users to maintain a mandatory clearance between the tablet and fingertips when not typing. Therefore, it is reasonable to believe that this extra constraint could produce wrist extension that accounted for the major portion of the wrist extension observed in our DESK position, regardless of which virtual keyboard was adopted.

### Wrist Deviation

The experimental data also demonstrated that typing with the tablet on the desk resulted in a substantially higher level of wrist ulnar deviation, unless the angled split keyboard was utilized. Our results are in agreement with the findings of Werth and Babski-Reeves [9]; they also observed greater ulnar deviation from typing tasks with the tablet on a desk, compared to a sofa scenario. In contrast, Young et al. [10] reported a similar level of wrist deviation between the lap and table postures while performing email tasks on tablets. It should be noted, however, that neither the experimental protocols nor the testing environments were the same among these studies. For example, participants in the studies by Young et al. [10] were required to type on tablets placed in cases that formed different tilt angles between the touchscreen and its support surface. This system could have created an extra demand on wrist extension [45], and thus may have accordingly changed the wrist posture in the coronal plane. Moreover, it is well known that the musculoskeletal system in humans is characteristic of its redundant properties, and therefore, the preferred hand positions with respect to the touchscreen can be achieved through fine-tuning the involved joint angles in different ways. Given the relatively constrained hand space afforded by the tablet compared to a conventional physical keyboard, tablet users might incline to assume a bed-typing posture that taxes postural muscles more (e.g. increase shoulder abduction and elbow flexion) (Fig 4A), instead of the one required to simultaneously deviate and extend the wrist to its extreme end. Likewise, from the perspective of wrist biomechanics, this may partially explain why the dominant (right) and non-dominant (left) hands acted dissimilarly in the LAP and BED positions.

It is generally believed that the split keyboard design can effectively mitigate the wrist deviation that users commonly experience while typing on a conventional QWERTY keyboard [22, 23, 48]. In the DESK position, we observed approximately 14 degrees and 6 degrees of median ulnar deviation from the STD and SPLIT virtual keyboards, respectively (Fig 3B); this is similar to the values reported by Rempel et al. [23]. When considering all examined positions of tablet usage, however, it was interesting to find that typing on the SPLIT virtual keyboard tended to encourage participants to maintain a neutral wrist posture without significant deviation in both the right (p = 0.385) and left hand (p = 0.062). On the contrary, a similar deviated level of wrist posture in the DESK setting was found on the STD layout and the WIDE key-spaced one (Fig 3B). This observation provides further support to Kim et al. [32]’s finding, where they found the wrist deviation level is generally not affected by the key size on a virtual keyboard.

### Elbow Flexion

In the present study, we observed that participants required to crunch in bed to type tended to flex their elbows further compared to those assigned to the other two positions, irrespective of the virtual keyboard design (Fig 4A). Since the upper extremities are complex kinematic chains, the level and range of motion of a particular body joint could be affected by adjacent joints. The reported average of 75 degrees of median elbow flexion was believed to be the result of the multiple task and physiological demands imposed by prolonged typing. During the experimental periods, we found that participants inclined to place the tablet close to their knees in order to reduce the viewing distance and allow their wrists and forearms to simultaneously rest on their thighs. However, placing the tablet either on the desk or in their laps resulted in a similar level of elbow flexion, with values generally lower than those seen in past studies examining laptop computers and workstation design [3, 4, 16, 45]. Nevertheless, Rempel et al. [24] did report comparable values in their keyboard design work. The differences in simulated tasks, experimental settings, and instrumentation likely contribute to the diverse measurements of elbow flexion seen in the literature. For example, typing on an internal smaller laptop keyboard has been shown to produce less mean elbow flexion than a full-sized external keyboard [3]. Furthermore, some researchers suggest that situating the keyboard away from the body has the tendency to lead to a posture with more elevated and flexed upper limbs, particularly when either the forearms are supported (such as our DESK scenario) [46] or when the use of a chair backrest is mandatory (such as the LAP scenario) [17]. These practices are generally accompanied by associated changes in shoulder abduction and forearm pronation [15]. Unfortunately, a lack of relevant measures in the present study made it difficult to confirm these speculations.

In regard to the effect of virtual keyboard design, Rempel et al. [24] have argued that the open angle of the physical keyboard is positively related to elbow separation. This ergonomic recommendation was at least partially sustained in our study, as we also observed a more neutral elbow posture when participants conducted text-entry tasks at the desk via the software implementation of the split keyboard design on the tablet (Fig 4A).

### Neck Flexion

Past studies on the effects of working configuration on posture in portable computers discovered that users have an increased neck flexion or head tilt while using the laptop positioned on the lap instead of a traditional desk [9, 16, 18]. Therefore, we expected to find similar results between the examined positions considering tablet usage. Surprisingly, neither working position nor virtual keyboard design had a meaningful influence on the 50^th^ percentile level of neck posture. This finding is consistent with the findings from previous work [9], in which the participants performed simple typing tasks with a tablet either placed on an adjustable desk or used while working on a sofa. For the neck flexion, they reported an angle of slightly lower than 10 degrees for both working conditions. Similarly, Young et al. [11] also did not observe significant differences in both neck flexion and head flexion between the desk and lap positions when participants executed a series of simulated tasks comprised of internet browsing, article reading, and email responding. The values of mean neck flexion reported from them, however, are considerably higher than what were found in the current study. This is likely attributable to the dissimilarity in angle definition, as well as instrumentation employed. Similar to what we did, Werth and Babski-Reeves [9] used electrogoniometers to monitor the relative changes in neck posture in the sagittal plane with the participant’s neutral neck posture as the zero baseline. In contrast, Young et al. [11] adopted an infrared three-dimensional motion system to measure the angle between the global vertical and the vector pointing from the C7 bony landmark to the lateral outer canthi. In addition, comparisons between reported neck-related flexion values among various research works should take into account the influence of normal curves of the spine, which could, to a certain extent, add an extra 15–25 degrees in some flexion measures around the neck/head regions [11, 34, 49]. To comprehensively describe the changes in the cervical spine biomechanics with respect to postural demands, future research may need to consider the interrelationships between neck/head flexion, viewing angle/distance, and neck-trunk angle in a systematic manner [8, 9, 11, 49].

One of the major contributions of the current study is that it quantitatively characterized posture variations during prolonged typing activities. Our results indicated that participants significantly increased their body movements in wrist extension/deviation, elbow flexion, and neck flexion after continuously typing on a tablet for an extended period of time (Table 2). The 23% increase in ROM averaged from all examined joints could be considered a consequence of self-protective motor strategies employed to relieve body discomfort and physiological strains due to the long task duration, as well as postural rigidity caused by non-neutral working positions. A lot of research has indicated that increased motor variability, manifesting in posture and movement, has the potential to reduce the risk of developing musculoskeletal disorders caused by sustained, repetitive works [50]. Some researchers further suggest that the acute pain or muscular fatigue induced during the experimental process may actually motivate the nervous system to increase motor variability to search for a better movement patterns with less inclination to aggravate discomfort or injury [50–52]. Our findings from the Borg CR-10 scale and posture measures generally supported the aforementioned arguments about the interrelationships between fatigue/pain and motor variability. Specifically, our data suggested that the body regions exhibiting less movement (neck and shoulder) throughout the experimental period tended to receive a higher discomfort rating compared to those (wrists and arms) feasible of varied joint excursions and muscle exertions (Tables 2 and 3).

### Perceivable usability

Our participants rated the WIDE layout with the highest SUS score when typing on a tablet in the common office setting, whereas the STD and SPLIT virtual keyboards gained better perceived usability than their counterparts in the lap and bed positions, respectively. From these results, it is apparent that the perceived usability of virtual keyboard design is influenced by the position of tablet usage. Kim et al. [32] used a traditional desk environment, and showed that a virtual keyboard with a key size less than 16 mm was less easy to use and had lower comfort ratings in upper-limb areas compared to those with bigger key sizes. In addition, Trudeau et al. [14] recently examined several virtual keyboard configurations during thumb typing scenarios in a common seated posture at work. Compared to the standard virtual keyboard, their participants expressed less discomfort while using the flat-split keyboard and simultaneously holding the tablet in grip postures. For tough typing, the angled split virtual keyboard evaluated in the present study also lead to less body discomfort in both the upper-limb and shoulder regions. Furthermore, both studies also found that use of the split-design virtual keyboard in the traditional office setting generally does not result in an inferior rating in perceived usability or task difficulty. However, it should be noted that the layouts of the split keyboards, as well as the typing methods employed in the two studies were relatively dissimilar.

These results need to be considered within the context of several potential limitations. First, we used a simulated text transcription task on a tablet to characterize the effects of working position and virtual keyboard design. The interactions between the users and the tablet during other non-typing tasks might cause different postural demands and physiological loadings on the musculoskeletal system. Second, to quantify the potential long-term effect of tablet usage, the participants were required to type continuously in the assigned working position throughout the entire 60-minute session. Although they were allowed to vary their postures within a certain range, in a natural setting it is still plausible that some users may prefer to type on the tablet with a posture substantially deviated from those observed in the present study. Lastly, this paper did not evaluate objective task performance, myoelectric signals from muscles pertinent to computer usage, or potential contact pressure imposed by the tablet or other artifacts in certain working environments. Additional insights elicited from those measures have shown to be valuable for detecting muscular fatigue in data-entry activities on desktop computers [21].

## Conclusions

The use of touch-screen media tablets in mobile environments is on the rise due to their portable and compact designs. The data suggest that users of tablets in non-traditional settings tend to assume non-neutral upper body postures to a level that could raise ergonomic concerns. As the usage duration increases, it causes substantial feelings of discomfort in certain body regions, and increases the chance of muscle fatigue. However, our results suggested that some of the negative health effects experienced could be mitigated by adequate design of the internal data input devices. Given the multidimensional nature of usability, it appears that the perceived usability of a particular virtual keyboard design is not only influenced by the stress imposed on the musculoskeletal system under varied usage positions, but also by the resulting task performance demands. To provide preemptive strategies to prevent potential ergonomic hazards in tablet usage, future research should consider the complex interrelationships between physiological loading, biomechanical demands, and desired task performances in a systematic manner.

## Abbreviations:

ANSI: American National Standards Institute
APDF: amplitude probability distribution function
MSDs: musculoskeletal symptoms or disorders
rANOVA: repeated analysis of variance
ROM: range of joint movement
WPM: words per minute
SUS: System Usability Scale
